# Follicular Tonsilitis and Idiophatic Peritonitis

**Published:** 1887-11

**Authors:** 


### *FOLLICULAR TONSILITIS AND IDIOPATHIC PERITONITIS.*

Dr. Fröhlich, in the *Deutsch Medicinal Ztg.*, No. 37, 1887, reports the following cases, which are of great interest, because they prove that follicular tonsilitis, which is usually regarded as a harmless disease, can become an object of great importance:

A military musician, L——, acquired a follicular tonsilitis; he had no fever, but the pulse was small and frequent, and the general health bad. The next day, temperature thirty-nine and one-tenth, pulse 108; meteorism and complaints of abdominal pains. There was also an enlargement of the spleen, rendering the condition like typhoid. But, next day, it was obviously seen that the man suffered from a peritonitis. In spite of energetic treatment with ice, calomel and camphor and benzoin injections, the condition aggravated, the weakness increased and the patient died the next day. The post-mortem examination showed a simple peritonitis without perforation, œdema pulmonum and swelling of the tonsils, without, however, the presence of membranes. The affection was considered to be a simple peritonitis. During the post-mortem examination, the author cut himself in a finger, and, in spite of energetic disinfection, he was attacked the next day with violent lymphangeitis and lympadenitis of his arm; high fever, and very bad general condition. He also experienced pain in the pharynx, and examination showed a follicular tonsilitis. The next day, the wife of the author also suffered from a follicular tonsilitis, and the same day, an assistant, who had sown up the cadaver, and also wounded his finger, acquired a follicular tonsilitis. It was now evident there was a connection between the infection of the fingers and the tonsilitis, and that the tonsilitis was so infectious that the wife had acquired it. It was nearly certain that the tonsilitis of the author and the assistant was caused by the peritoneal pus from the cadaver, and that both affections were caused by the same micro-organism. The author, his wife and the assistant were successfully cured. The author refers to some cases in literature of similar affections, but the connection is not so certainly established in any of these as in this case. Bacillary examinations were not made, because the musician had been already buried when the chain of events became interesting.—*Journal of Laryngology and Rhinology*.

